# Complete genome sequence of *Glycocaulis abyssi* type strain LMG 27140, a prosthecate bacterium isolated from an abyssal hydrothermal vent

**DOI:** 10.1128/mra.00984-24

**Published:** 2024-10-14

**Authors:** Breah LaSarre, Eva M. Kierstead, Amelia M. Randich

**Affiliations:** 1 Department of Plant Pathology, Entomology, and Microbiology, Iowa State University, Ames, Iowa, USA; 2 Department of Biology, University of Scranton, Scranton, Pennsylvania, USA; DOE Joint Genome Institute, Berkeley, California, USA

**Keywords:** Alphaproteobacteria, Caulobacterales, *Maricaulaceae*, prosthecate, hydrothermal vent

## Abstract

*Glycocaulis abyssi* is a dimorphic, prosthecate member of the family *Maricaulaceae*, order Caulobacterales, in the class Alphaproteobacteria, originally isolated from an abyssal hydrothermal vent. Here, we report the complete genome sequence of type strain LMG 27140^T^ (MCS33^T^) obtained using Oxford Nanopore technology.

## ANNOUNCEMENT

*Glycocaulis abyssi* was isolated from seawater sampled at 1,762 m depth near a black smoker of the Juan de Fuca ridge (1). It was originally characterized as a prosthecate, dimorphic bacterium of the family *Hyphomonadaceae*, with the highest 16S rRNA similarity, 94.2%, to *Maricaulis maris* ATCC 15285^T^ and *Oceanicaulis alexandrii* C116018^T^ (2). *Glycocaulis, Maricaulis,* and *Oceanicaulis* have since been reassigned to the family *Maricaulaceae* (3). A complete genome sequence exists for one other *Glycocaulis* species (*Glycocaulis alkaliphilus* 6B-8) (4) but not this unique deep-sea member. Although *G. abyssi* grows optimally at 30°C like other *Glycocaulis* species, its isolation site and lack of phospholipids suggest adaptations for living in a marine benthic zone (2), which the genome sequence will aid in identifying.

One LMG 27140 colony from a dimethylsulfoxide (DMSO) stock (propagated from lyophilisate from BCCM/LMG) was grown in 25 mL Marine Broth (BD) for 2 days at 30°C, 220 rpm to early stationary phase. Cells were washed once in Phosphate-buffered Salin (PBS) and resuspended in DNA/RNA Shield (Zymo Research) before shipment to Plasmidsaurus for DNA isolation (Quick-DNA Miniprep Plus Kit, Zymo), library preparation [Rapid Barcoding Kit 96 V14, Oxford Nanopore Technologies (ONT)], and long-read sequencing (PromethION P24, R10.4.1, flow cell). Bases were called in the super-accurate mode (ont-doradod-for-promethion v7.1.4, model dna_r10.4.1_e8.2_400bps_sup@v4.2.0), with a *Q*-score cutoff of 10, producing 176,603 reads (*N*_50_: 8,464 bp). Raw reads were filtered to eliminate the worst 5% of reads and reads < 1,000 bp (Filtlong v0.2.1, https://github.com/rrwick/Filtlong; --min_length 1000 --keep_percent 95), yielding 121,059 filtered reads (*N*_50_: 8,972 bp). Following read subsampling into six subsets [Trycycler v.0.5.4 (5) “subsample” function; target genome size, 3.5 Mbps], each subset was assembled with Miniasm/Minipolish v0.3-r179/0.1.3 (6, 7), Raven v1.8.3 (8), and Flye v2.9.3 (9). The 18 assemblies were merged into a consensus genome using the full Trycycler pipeline according to the online documentation (https://github.com/rrwick/Trycycler/wiki), followed by consensus polishing using Medaka v1.11.3 (https://github.com/nanoporetech/medaka; ONT). The chromosomal contig was rotated to start at the *dnaA* gene using the fixstart function in Circlator v1.5.5 (10). The plasmid contig was manually rotated to start at the *repC* gene predicted by bakta v1.9.3 (11). Default parameters were used unless noted otherwise.

The LMG 27140 genome comprises two contigs, a circular 2,883,807-bp chromosome and a 169,948-bp circular plasmid, with 63.35% average GC content and 245× average sequencing depth [per Quast v5.2.0 (12)], and estimated 100% completeness with 0.12% contamination [per CheckM2 v1.0.2 (13)]. LMG 27140 has 2,894 total protein-coding genes, similar to its close relatives, but is distinct in having a plasmid (Table 1). The genome was annotated with the NCBI Prokaryotic Genome Annotation Pipeline during GenBank submission.

**TABLE 1:** Genome characteristics of *G. abyssi* compared to other complete *Glycocaulis* and *Maricaulaceae* genomes

	*G. abyssi* LMG 27140	*G. alkaliphilus* 6B-8	*Maricaulis maris* ATCC 15285T	*Oceanicaulis* sp. (metagenomic)
Assembly accession no.	CP163421 –CP163422	NZ_CP018911.1	NZ_AP027270.1	CP157796.1
Chromosome size (bp)	2,883,807	3,019,659	3,142,398	3,078,920
Plasmid count (size)	1 (169,948 bp)	0	0	0
Total genes	2,967	2,892	3,008	3,032
Protein	2,894	2,837	2,933	2,969
rRNA	3	3	6	3
tRNA	44	43	47	43
Other RNA	4	3	4	4
Total pseudogenes	22	6	18	13

## Data Availability

The complete genome sequence has been deposited at NCBI GenBank under the accession numbers CP163421.1 (chromosome), CP163422.1 (plasmid pGa27140), BioProject accession number PRJNA1138649, and BioSample accession number SAMN42730192. The raw sequences have been deposited in the SRA under the accession number SRR29931579.
